# Infection by chikungunya virus modulates the expression of several proteins in *Aedes aegypti* salivary glands

**DOI:** 10.1186/1756-3305-5-264

**Published:** 2012-11-15

**Authors:** Stephane Tchankouo-Nguetcheu, Edouard Bourguet, Pascal Lenormand, Jean-Claude Rousselle, Abdelkader Namane, Valerie Choumet

**Affiliations:** 1 Unité de Génétique Moléculaire des Bunyavirus, 25 rue du Dr Roux, 75724, Paris cedex 15; 2 Unité de Biochimie et de Biologie Moléculaire des Insectes, Institut Pasteur, 28 rue du Dr Roux, 75724 cedex 15, Paris, France; 3 Plate-forme protéomique PF5, Institut Pasteur, 28 rue du Dr Roux, 75724 cedex 15, Paris, France; 4 present address: Unité Interactions Moléculaires Flavivirus-Hôtes, Institut Pasteur, 25, rue du Dr Roux, 75724 cedex 15, Paris, France

**Keywords:** *Aedes aegypti*, Chikungunya virus, Mosquito salivary gland, Proteomics, Two-dimensional gel electrophoresis, Mass spectrometry

## Abstract

**Background:**

Arthropod-borne viral infections cause several emerging and resurging infectious diseases. Among the diseases caused by arboviruses, chikungunya is responsible for a high level of severe human disease worldwide. The salivary glands of mosquitoes are the last barrier before pathogen transmission.

**Methods:**

We undertook a proteomic approach to characterize the key virus/vector interactions and host protein modifications that occur in the salivary glands that could be responsible for viral transmission by using quantitative two-dimensional electrophoresis.

**Results:**

We defined the protein modulations in the salivary glands of Aedes aegypti that were triggered 3 and 5 days after an oral infection (3 and 5 DPI) with chikungunya virus (CHIKV). Gel profile comparisons showed that CHIKV at 3 DPI modulated the level of 13 proteins, and at 5 DPI 20 proteins. The amount of 10 putatively secreted proteins was regulated at both time points. These proteins were implicated in blood-feeding or in immunity, but many have no known function. CHIKV also modulated the quantity of proteins involved in several metabolic pathways and in cell signalling.

**Conclusion:**

Our study constitutes the first analysis of the protein response of *Aedes aegypti* salivary glands infected with CHIKV. We found that the differentially regulated proteins in response to viral infection include structural proteins and enzymes for several metabolic pathways. Some may favour virus survival, replication and transmission, suggesting a subversion of the insect cell metabolism by arboviruses. For example, proteins involved in blood-feeding such as the short D7, an adenosine deaminase and inosine-uridine preferring nucleoside hydrolase, may favour virus transmission by exerting an increased anti-inflammatory effect. This would allow the vector to bite without the bite being detected. Other proteins, like the anti-freeze protein, may support vector protection.

## Background

Infectious diseases are one of the leading causes of death worldwide, with about 17% of deaths caused per year (WHO). Among these diseases, arthropod transmitted ones are major threats to the health of man and animals, and play an important role in the world economy [1]. The pathogens involved include parasites, such as *Plasmodium* or lymphatic filariosis, and arboviruses, like chikungunya, dengue, Rift Valley, yellow fever, Japanese Encephalitis and West Nile viruses. Traditional means of controlling the spread of arbovirus infections include the vaccination of susceptible vertebrates and mosquito control. However, in many cases such measures are either unavailable or ineffective. To successfully implement a strategy to block the virus at the insect stage, further knowledge of virus/vector interactions is required. Studies in this field may identify new genes and possible targets for altering virus/vector interactions.

For an arthropod to serve as an efficient arbovirus vector, three parameters are defined. The arthropod must ingest sufficient viremic blood to infect gut cells. After entering gut cells, sufficient viral replication must occur so that the virus can enter the hemocoel and infect other tissues such as salivary glands. Multiplication at this latter site ensures transmission within the saliva during a mosquito’s bite [2]. The saliva of arthropods contains a complex mixture of proteins and peptides, such as sugar-degrading enzymes (glycosidases), antimicrobials and components with anti-hemostatic, angiogenic, anti-inflammatory and immunomodulatory properties [3-5].

Amongst the various blood-feeding arthropods, the *Ae. aegypti* mosquito is one of the most anthropophilic and cosmotropical mosquito vectors. It has been implicated in several outbreaks of dengue, chikungunya, yellow fever and other arboviruses. The recent sequence of the *Ae. aegypti* Liverpool strain genome facilitated gene identification in this species [6]. Experimental evidence of mosquito gene function in response to pathogens is also now becoming available through the use of RNA-based and protein-based approaches. Certain vector proteins that react to vector/pathogen or vector/endosymbiont interactions have been identified already [7-10]. Their role in vector defence against aggression, or in pathogen transmission, has been discussed [7,9-12]. In contrast to mRNA-based approaches, proteomics is a tool that detects changes in protein expression and modification, and thereby provides comprehensive information related to induced changes in the infection.

In this work, we chose to analyze the interaction between chikungunya virus (CHIKV) and *Ae. aegypti* salivary glands. CHIKV is a mosquito-borne emerging pathogen that has a major health impact in humans, and causes fever, headache, rash, nausea, vomiting, myalgia, and arthralgia. The virus is indigenous to tropical Africa, but there have been reports of widespread outbreaks in parts of South East Asia and several of its neighbouring islands in 2005–07 and in Europe in 2007 [13]. Furthermore, positive cases have been confirmed in the United States in travellers returning from known outbreak areas [14]. Currently, there is no vaccine or antiviral treatment against CHIKV. This virus is an alphavirus of the Togaviridae family; enveloped, with a 70 nm diameter capsule [15] and a single-stranded linear RNA genome of positive polarity, approximately 11.8 Kb long [16]. CHIKV is known to have a short extrinsic incubation period in *Ae. aegypti* mosquitoes and is present in the saliva as early as 4 days post-infection (DPI). In our approach, we aimed to identify early modulations of salivary gland responses to CHIKV. We chose two time points post-infection and determined by two dimensional electrophoresis coupled to tandem mass spectrometry (2DE-MS/MS) which proteins were affected by the presence of the virus.

This study provides interesting data that increase our understanding of CHIKV/salivary gland interactions and will allow the design of new approaches to block virus transmission at the *Ae. aegypti* salivary gland level. It also raises important questions concerning the role of the modulated saliva proteins relative to the transmission of chikungunya virus.

## Methods

### Mosquitoes

*Ae. aegypti* (PAEA strain) mosquitoes were maintained at 28±1°C under 80% relative humidity with a light/dark cycle of 16 h/8 h. Larvae were reared in pans containing a tablet of yeast in 1 L of tap water. Adults were provided with 10% sucrose solution *ad libitum*.

### Viruses

The CHIKV 06.21 strain isolated in November 2005 from a new-born male in La Reunion who presented meningo-encephalitis symptoms was used for all experiments [17]. This strain contained an A→V change at position 226 in the E1 glycoprotein (E1-226V). Stock virus was produced following passages on *Aedes albopictus* C6/36 cells then harvested and stored at −80°C in aliquots as described [18]. The titre of the frozen stock virus was estimated to be 10^9^ plaque-forming units (PFU)/mL.

### Oral infections of mosquitoes and dissections

Seven day-old female mosquitoes were deprived of sucrose for 24 h prior to the infectious blood meal. They were then allowed to feed for 15 min through chicken skin membranes covering glass feeders maintained at 37°C. The infectious blood meal was comprised of two thirds washed rabbit erythrocytes, one third viral suspension, with ATP as a phagostimulant at a final concentration of 5x10^-3^ M. The infectious blood was at a titre of 10^7.5^ PFU/ml CHIKV 06.21. Three independent infections were performed.

### Salivary gland collection

Salivary glands were collected from female mosquitoes, infected or not infected with CHIKV, 3 and 5 days after the blood meal. They were placed into a tube containing 100 μl of 150 mM sodium chloride (NaCl) and protease inhibitors (Complete, Roche). Samples were stored at −80°C until use.

### Reverse transcription and quantitative PCR (RT-q PCR)

Total RNA from mosquitoes or salivary glands was extracted using the Nucleospin® RNA II kit (Macherey-Nagel) according to the manufacturer's instructions. RNA was eluted in 40 μl of RNAse-free H_2_0 by centrifugation at 11,000 g for 1 min.

Synthetic RNA transcript for CHIKV was generated to construct a standard curve. The targeted region in the CHIKV sequence was amplified by PCR and ligated into the pCR II TOPO vector (Invitrogen). The plasmid was then linearized using the EcoRI restriction enzyme and purified using the QIAquick PCR purification kit. RNA transcripts were prepared *in vitro* using the RiboMAX™ Large Scale RNA Production Systems (Promega) appropriate for either SP6 or T7 RNA polymerase. The transcript size was 1,356 bp. Residual DNA was eliminated by several DNAse treatments (Turbo DNA-free (Ambion)). After quantification by spectrophotometery, RNA transcript solutions were stored at −80°C.

One-step reverse transcription quantitative PCR (RT-qPCR) was performed using the Power Sybr Green RNA-to-Ct one step kit (Applied Biosystems). CHIKV primers were selected in the E2 structural protein coding region: sense Chik/E2/9018/+ (CACCGCCGCAACTACCG); anti-sense Chik/E2/9235/- (GATTGGTGACCGCGGCA).

RT-qPCR was performed using Applied Biosystem’s Fast Real-Time PCR 7500 System with the v.2.0.1 7500 software. The thermal cycling conditions comprised: a reverse transcription step at 48°C for 30 min, an inactivation step of RT/RNAse enzyme at 95°C for 10 min followed by 40 cycles of 95°C 15 s and 60°C 1 min, a final denaturation step where the temperature was increased from 60°C to 95°C during 20 min and a step of 15 sec at 95°C. Signals were normalized to the standard curve using serial dilutions of RNA synthetic transcripts. Using ΔC_t_ analysis, normalized data were used to estimate the transcript copy number in infected mosquitoes.

### Immunofluorescence

After dissection, salivary glands were placed on a slide. PBS was removed and salivary glands were fixed in 4% paraformaldehyde for 1 h, dried and kept at 4°C until use. For indirect immunofluorescent assay (IFA) experiments, salivary glands were rehydrated in PBS for 3 x 5 min, and then incubated for 15 min with Triton X100 (0.2%). They were washed again with PBS (3 x 5 min) and incubated for 30 min with PBS that contained 1% BSA. The slides were drained and incubated overnight at 4°C with Cy3 conjugated anti-chikungunya E 3E4 protein diluted 1:500 in PBS, then washed with PBS (3 x 5 min) under shaking. The actin network was stained with Phalloidin Alexafluor 488 (Invitrogen) (diluted 1/40 in PBS). After washing, a drop of Prolong gold antifade (Invitrogen) was dropped onto each slide and a coverslide was placed on top. All preparations were examined by confocal microscopy (Zeiss LSM 510 Meta and TCS SP5 Leica Microsystems).

### Preparation of salivary gland protein extracts

3 and 5 days after feeding, pools of 200 salivary glands were dissected in 100 μl PBS that contained protease inhibitors (Complete, Roche Diagnostics). These were kept at −80°C until use. Salivary glands were disrupted by ultrasound (Cup Horn, Sonics & Material) for 20 min with 2 sec pulse on and 2 sec pulse off, at the maximum amplitude. Salivary gland homogenates were then centrifuged for 30 min at 130,000 g and proteins were quantified using the BCA protein assay (Pierce). Aliquots of salivary gland proteins were then lyophilized either for immediate use or for storage at −80°C. The protein concentration of salivary gland extracts (SGE) was determined by spectrophotometry using a Nanodrop ND-1000 Spectrophotometer (Nyxor Biotech).

SGE samples were lyophilized (Christ, ALPHA 1–4 LD model; vacuum pump: Vaccubrand, MZ2-SE 220V 60 Hz model) and either resuspended in 20 μl of sterile distilled water for immediate use or stored dried at −80°C.

### Two-dimensional gel electrophoresis

Three 130 μg samples were analyzed for each of infected and non-infected salivary glands. The first dimension of electrophoresis was by isoelectric focusing and the second dimension by SDS/polyacrylamide gel electrophoresis. Samples containing 130 μg (30 μl) of SGE were placed on ice for 20 min, then vortexed and centrifuged for 5 sec. 1.2% benzonase (Sigma) was added and samples incubated for 1 min at 4°C. They were then mixed with 320 μl of rehydration solution containing 1% of pH 3–10 carrier ampholytes (Invitrogen), 7M urea (Bio-Rad), 2M thiourea (Sigma), 4% CHAPS (Sigma), 100 mM DTT (Bio-Rad) and 0.002% bromophenol blue (Sigma), vortexed and centrifuged for 5 sec. In-gel rehydration was performed using strip holders, the strip (18 cm, pH 3–10 NL, GE Healthcare) being covered with 3–4 ml of oil (Cover Fluid, GE Healthcare). The first dimension separation protocol was conducted stepwise, according to the following protocol: an active rehydration for 5 h at 30 V, 500 V for 2 h, 1000 V for 30 min, 1500 V for 30 min, 2500 V for 30 min, 5000 V for 10 h and 8000 V for 2 h. 50 μA maximum were applied to the strips at 20°C (Ettan IPGphor III, GE Healthcare). The strips were then equilibrated for 15 min in buffer containing 6 M urea (Bio-Rad), 50 mM Tris–HCl pH 8.8 (Sigma), 30% glycerol (Prolabo), 2% SDS (Prolabo), 0.002% bromophenol blue with 1% DTT (Bio-Rad) for the first step and 2.5% iodoacetamide (Sigma), for the second step. For the second dimension, the strip was loaded on a 1 mm-thick 12% SDS PAGE gel with no stacking gel, and embedded into the SDS PAGE gel using 1% agarose. Gel electrophoresis was performed for 7 h 45 min at 45 mA and 200 V/gel. The slab gels were then stained with SYPRO Ruby (Invitrogen).

### Analysis of gel patterns

SYPRO Ruby stained gels were scanned with a Typhoon 9400 variable-mode imager (GE Healthcare) and compared using ImageMaster 2D Platinum software (GE Healthcare). All spots identified by the software were verified by eye. All spots reported had at least a 1.8 fold intensity (normalized volume) difference between infected and non-infected samples, and were all statistically significant according to the statistical tool built into the software (Anova test, with p<0.05).

### Protein preparation for mass spectrometry

After SYPRO Ruby staining, all visible gel bands were excised using the ProPic Investigator robotic work station (Genomic Solutions, Ann Arbor, MI), then plugs were collected in a 96-well plate. Proteins were reduced, alkylated, and digested overnight with porcine-modified trypsin (Promega Sequencing grade, ratio 1:100) at 37°C using the ProGest Investigator (Genomic Solutions, Ann Arbor, MI,USA). The trypsin digests were desalted with C_18_ tips (μZipTip, Millipore). Peptides were directly eluted, using the ProMS Investigator (Genomic Solutions, Ann Arbor, MI,USA), onto a 96-well stainless steel MALDI target plate (Applied Biosystems/MDS SCIEX) with 0.5 μl of ∝-Cyano-4-Hydroxy Cinnaminic Acid (2.5 mg/ml in 70% acetonitrile, 30% H_2_O, 0.1% trifluoroacetic acid).

### Mass spectrometry analysis

Raw data for protein identification was obtained using the 4800 Proteomics Analyzer (Applied Biosystems/MDS SCIEX, Framingham, MA, USA) and was analyzed using the GPS Explorer 2.0 version 3.6 software (Applied Biosystems/MDS SCIEX, Framingham, MA, USA). For positive ion reflector mode spectra, 2000 laser shots were averaged. For monoisotopic (MS) calibration, autolysis trypsin peaks ([M + H]^+^ = 842.5100 and 2211.1046) were used as internal calibrators. MS peak masses were automatically determined within the 800–4000 Da range with a signal to noise ratio minimum set to 30. Up to 25 of the most intense ion signals were selected as precursors for MS/MS acquisition, excluding common trypsin autolysis peaks and matrix ion signals. In MS/MS positive ion mode, 4000 spectra were averaged; the collision energy was 2 kV, the collision gas was air, and the default calibration was set using Glu^1^-fibrinopeptide B ([M + H]^+^ = 1570.6696) spotted onto 13 positions on the MALDI target. Combined Peptide Mass Fingerprint (PMF) and MS/MS queries were performed using the MASCOT 2.1 search engine (Matrix Science Ltd., London, UK) embedded into GPS Explorer software on the NCBInr database (downloaded on 2010 01 19; 10348164 sequences; 3529470745 residues) with the following parameter settings: 50-ppm mass accuracy for MS, trypsin cleavage with one missed cleavage allowed, carbamidomethylation set as fixed modification, oxidation of methionines and formation of Pyro-Glu (N-term E and N-term Q) allowed as variable modifications, and MS/MS fragment tolerance set to 0.3 Da. Protein hits with MASCOT protein score ≥ 83 and peptide hits with Ionscore ≥ 53 and a GPS Explorer protein confidence index ≥ 95% were used for further manual validation.

## Results and discussion

### Follow-up of CHIKV infections in orally infected *Ae. aegypti* females at 3 and 5 DPI : IFA and RT-qPCR

**Figure 1:**
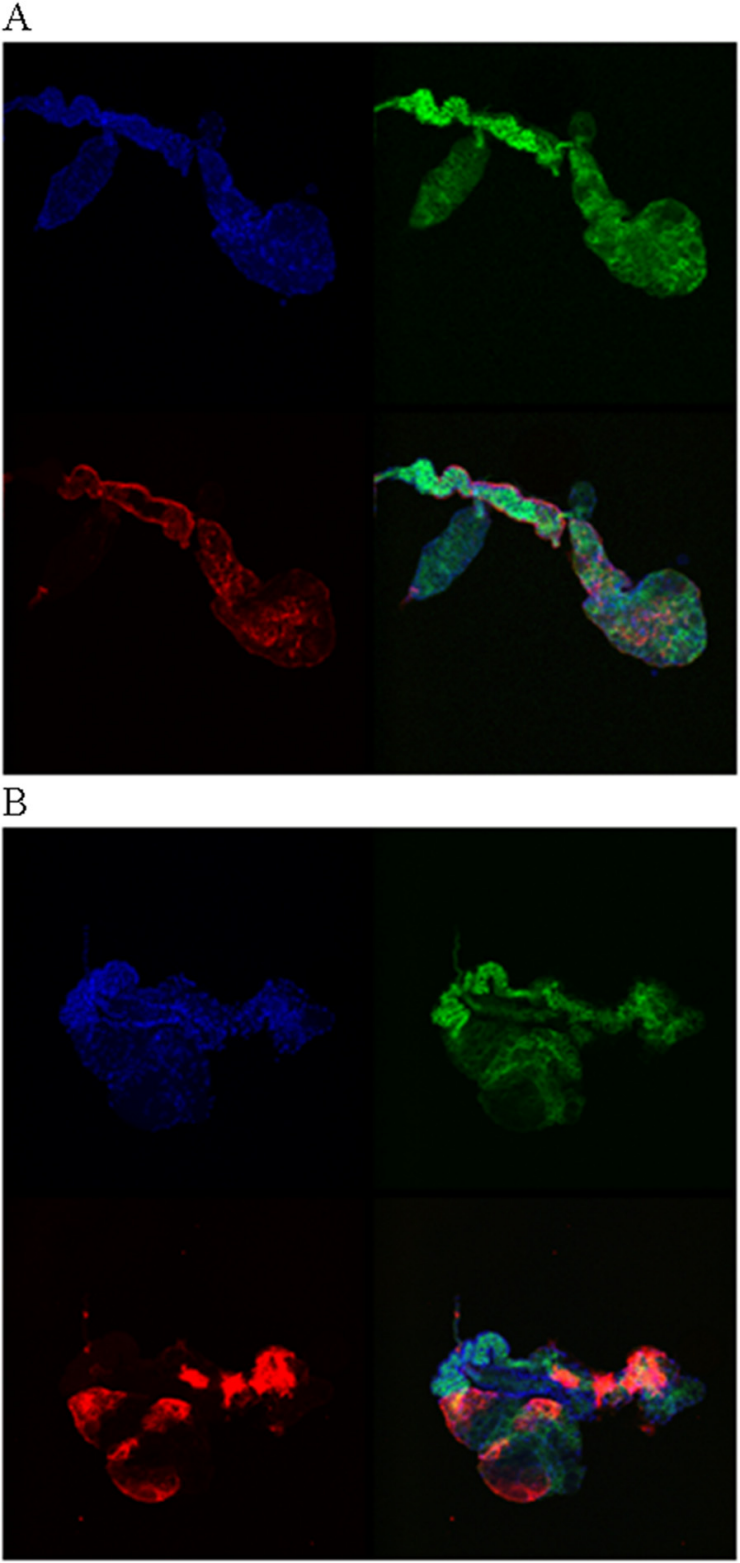
**Distribution of CHIKV in 3DPI and 5DPI *Ae. aegypti *salivary glands. **Salivary glands were dissected at 3 DPI (**A**) and 5 DPI (**B**). CHIKV labelled in red was identified using 3E4 anti-CHIKV according to the protocol described in the Methods section. Nuclei were labelled in blue using DAPI. Alexafluor 488-labelled phalloidin was used to label the actin network in green. A superimposition of all labelling is also shown.

**Figure 2:**
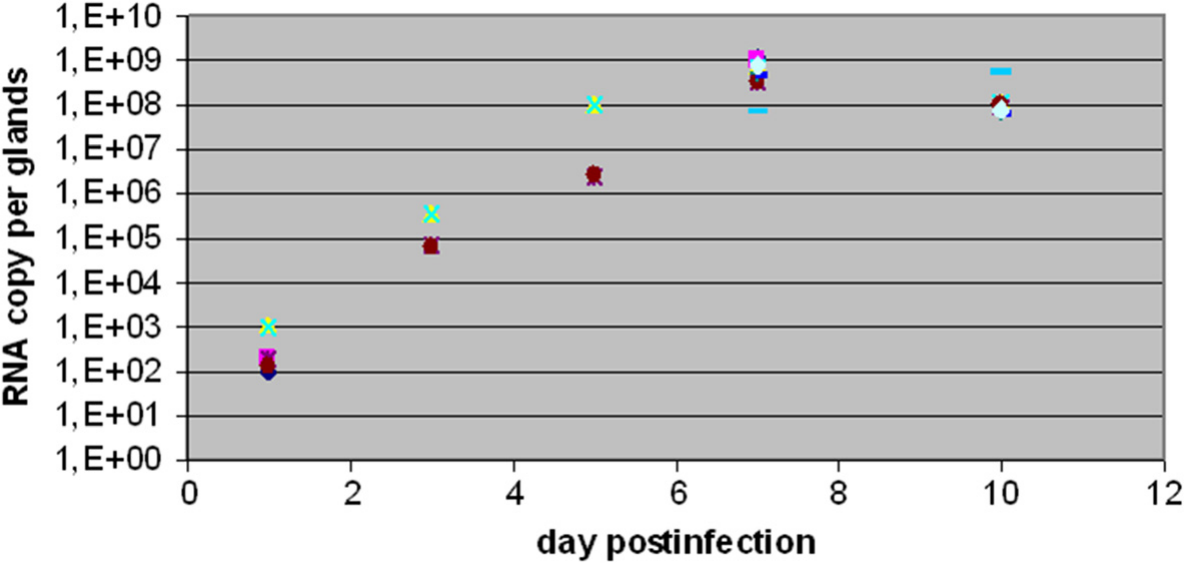
**Quantification of CHIKV RNA by RT-qPCR in infected salivary glands. **The viral RNA copy number of CHIKV was measured at 1, 3, 5, 7 and 10 DPI.

Depending on the mosquito strain, CHIKV is found in the salivary glands 2 to 4 days after acquisition [19]. To analyze salivary gland infection at different times post-infection, we used two different approaches: i) visualization of the distribution of virion particles by IFA, and ii) quantification of viral RNA. Figure 1A and B show the distribution of CHIKV in *Ae. aegypti* 3 and 5 DPI. CHIKV particles were revealed in the salivary gland lateral lobes at 3 DPI, whereas they had invaded the whole salivary glands by 5 DPI. RNA copy number was measured by RT-qPCR at 1, 3, 5 and 10 DPI (Figure 2). In agreement with IFA observations, the CHIKV RNA copy numbers increased from 3 DPI to 5 DPI, almost reaching a plateau between these two time-points. Then RNA copy number remained constant until 10 DPI.

### Two-dimensional gel electrophoresis (2DE) analysis of differential expression in CHIKV-infected salivary glands

**Figure 3:**
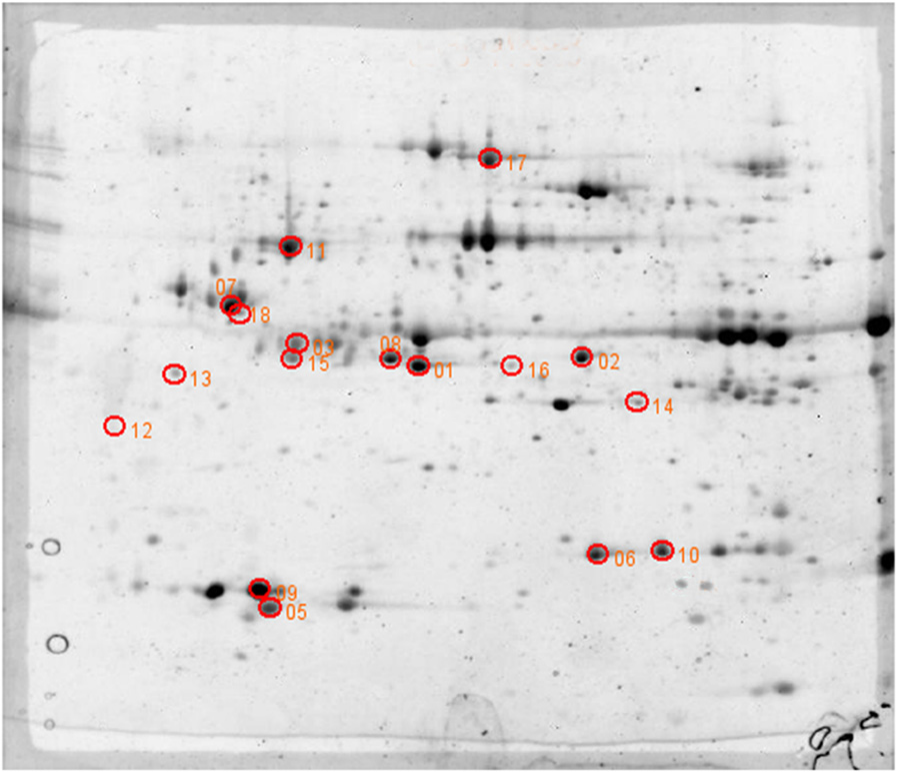
**Spots up-regulated at 3DPI in CHIKV-infected salivary glands of *Ae. aegypti. ***130 μg of salivary gland extract from 3 DPI CHIKV-infected mosquitoes and control mosquitoes were loaded onto 3–10 NL immobilins (18 cm). The immobilins were then deposited on the top of 12% SDS-PAGE gels. Spots were revealed using SYPRO Ruby. Gel profiles were compared using Image Master Platinum software. The spots that were found up-regulated at 3DPI are indicated by circles (fold change>1.8; Anova<0.05).

**Figure 4:**
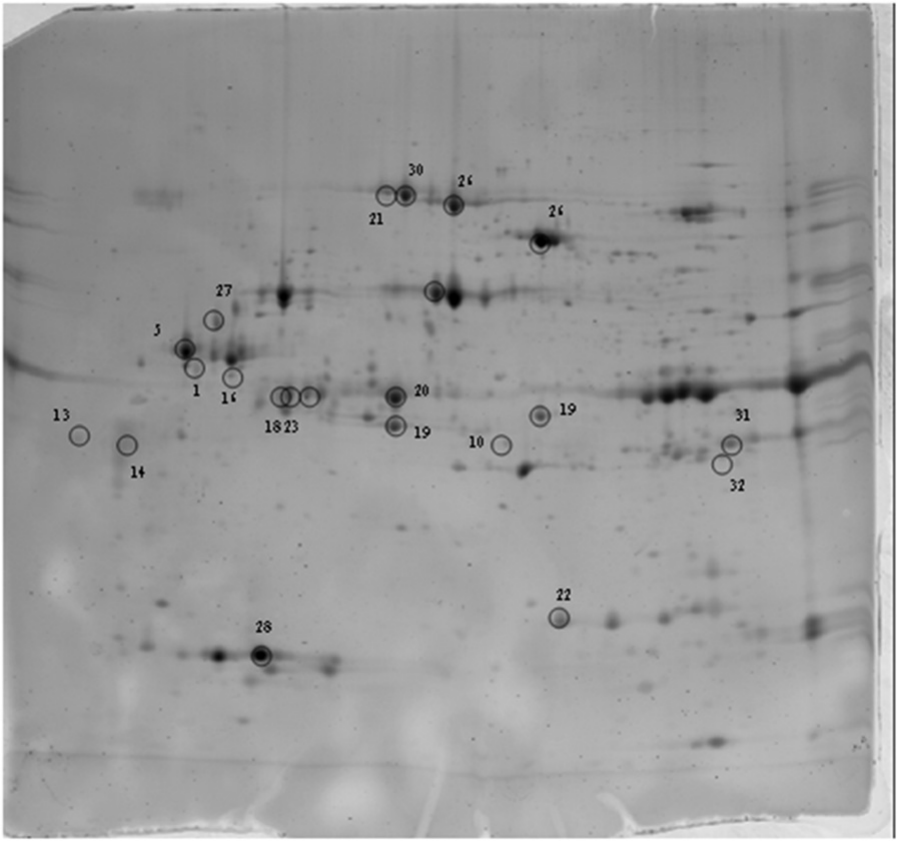
**Spots up-regulated at 5DPI in CHIKV-infected salivary glands of *Ae. aegypti. ***130 μg of salivary gland extract from 5 DPI CHIKV-infected mosquitoes and control mosquitoes were loaded onto 3–10 NL immobilins (18 cm). The immobilins were then deposited on the top of 12% SDS-PAGE gels. Spots were revealed using SYPRO Ruby. Gel profiles were compared using Image Master Platinum software. The spots that were found up-regulated at 5DPI are indicated by circles (fold change>1.8; Anova<0.05).

Three independent infections were performed for each time point, in parallel with three controls where artificial feeding was carried out with non-infected blood. Pools of 200 salivary glands were collected after each experiment and the same amount of protein extract from each pool (130 μg) was used for 2DE experiments. For each type of salivary gland, three gels were run according to the experimental protocol (see Materials and Methods). Images of gels showing control and 3 DPI and 5 DPI CHIKV-infected salivary gland extract profiles are shown in Additional file 1: Figure S1. Analysis of gels with 2D Image Platinum software allowed the detection of 250 to 330 spots per gel. Two analyses were performed on the gels: i) a comparison of control profiles with 3 DPI CHIKV-infected salivary glands; ii) a comparison of controls with 5 DPI CHIKV-infected samples (control/CHIKV 5DPI). A total of 55 variant spots were excised from the gels, digested by trypsin and analyzed by MALDI-TOF/TOF mass spectrometry (Figure 3; Figure 4; Additional files 2 and 3: Figure S2 and Figure S3; Additional file 4: Table S1). For the control/CHIKV 3DPI analysis, 17 spots were identified (Figure 3), and for the control/CHIKV 5DPI analysis, 21 (Figure 4). Several spots corresponded to the same protein, indicating that posttranslational modifications were occurring.

Lists of the proteins identified from these spots by mass spectrometry are shown in Additional file 4: Table S1, Additional file 5: Table S2, Additional file 6: Table S3 and Additional file 7: Table S4. These tables indicate the protein identification number, their spot number in the corresponding gel, the fold modulation of protein expression observed in infected versus control gels and the putative localization and function of the proteins.

Table 1 shows the modulation triggered at each time point for each identified protein from all of the gel data comparisons listed according to their putative function. Analyses of the control/CHIKV 3DPI and control/5DPI comparisons show that 13 proteins are modulated at 3DPI and 19 at 5 DPI (Table 1). The virus modulates 10 proteins at both time points, most of them in the same manner (up-regulation), and two proteins are differentially expressed at 3 DPI and at 5 DPI. The level of 13 proteins is modified at only one of the two time points, 3 proteins at 3DPI and 10 proteins at 5DPI.

**Table 1:** List of salivary gland proteins modulated in presence of CHIKV

**Protein/function**	**Identification Genebank**	**3DPI/NI**	**5DPI/NI**
**Housekeeping proteins**			
*Intracellular cell signalling processes*			
ran	gi|108874624	Nd in NI SG	Nd in NI SG
Fibrinogen and fibronectin *	gi|108883990	−6	+ 2.3
*Nucleotide metabolism*			
adenosine deaminase	gi|108878609		+ 2.1
inosine-uridine preferring nucleoside hydrolase *	gi|157113519	+2.1/4.6	+ 1.9/2.4
*Fatty acid metabolism*			
cyclohex-1-ene-1-carboxyl-CoA hydratase, putative	gi|108880435	+4	
*Proteins involved in cytoskeleton*			
beta-1 tubulin	gi|111035024		−2
*Anti-oxydant related*			
malic enzyme	gi|108883625		−2.3
protein disulfide isomerase	gi|157107430		−2
**Secreted protease inhibitor**			
SERPIN1 protein precursor, putative serpin [Aedes aegypti] 47 kDa	gi|108881296		−10
SERPIN1 protein precursor, putative serpin 41 kDa	gi|108881297		+ 2.7
putative serpin *	gi|18568304	+ 2	+ 2 .3
**Immunity related protein**			
Angiopoietin-like protein variant [Aedes aegypti]°	gi|94468352	+4.5	
**Blood feeding**			
Apyrase, putative [Aedes aegypti]*	gi|157113141	**+** 3.2/3.4	+ 1.9/2.2/2.5
30 kDa salivary gland allergen Aed a 3 [Aedes aegypti]*	gi|2114497	−3	+ 3
putative 30 kDa allergen-like protein	gi|18568322		Nd in NI SG
**Unknown**			
Antifreeze protein, putative [Aedes aegypti]	gi|157103422	+ 2.2	+ 1.9
Venom allergen [Aedes aegypti]	gi|18568284		+ 4.1
putative 16.9 kDa secreted protein	gi|18568330	+ 1.9	
Putative 34 kDa family secreted salivary protein	gi|94468642	+2.1/7	+ 2.1/2.2
62 kDa family	gi|108883987		+ 1.9
Conserved hypothetical protein	gi|108880897		+2
Conserved hypothetical protein	gi|108883988	+2.2/4	+2
short salivary D7 protein	gi|157115994	+1.9	+2.5

*proteins for which a modulation of gene expression was observed in dengue-infected salivary glands, ° proteins for which a modulation of gene expression was observed in blood-fed salivary glands, SG: salivary glands, Nd: not detected, NI: non infected.

We then investigated the modulation of protein expression related to putative function.

### Housekeeping proteins

#### Proteins involved in nucleotide metabolism

We found that denosine deaminase (ADA) was up-regulated in 5DPI SGE (Table 1). ADAs catalyze the deamination of adenosine to inosine and of deoxyadenosine to deoxyinosine. The ADA gene can be expressed in various mosquito tissues, but it is highest in salivary glands [20]. Secretion of ADA during blood feeding by *Ae. aegypti* has been demonstrated [21]. It has been suggested that ADA activity in *Ae. aegypti* removes adenosine, a molecule associated with both the initiation of pain perception and the induction of mast cell degranulation in vertebrates, and produces inosine, a molecule that potently inhibits the production of inflammatory cytokines. Thus, we hypothesize that a higher level of ADA in saliva may improve blood feeding in CHIKV-infected females.

Interestingly, we observed that the level of inosine-uridine preferring nucleoside hydrolase was increased in 3DPI SGE (Table 1). This enzyme catalyses the hydrolysis of all of the commonly occuring purine and pyrimidine nucleosides into ribose and the associated base, but has a preference for inosine and uridine as substrates. A salivary purine hydrolase activity has been reported in *Ae. aegypti*, and saliva is actually one of the richest sources of this enzyme [22]. It may actually play a role in blood feeding in combination with ADA that is present in the same mosquito. Effectively, it would then complete the catabolism of the vertebrate host adenosine to hypoxanthine, destroying a mediator of mast cell degranulation as mentioned above.

#### A protein involved in fatty acid metabolism

Cyclohex-1-ene-1-carboxyl-CoA hydratase is a member of the enoyl-CoA hydratase/isomerase family and displays catalytic activity. We found its level was increased in 3DPI SGE (Table 1). This family of proteins plays a particularly important role in the metabolism of unsaturated fatty acids. A significant down-modulation in the expression of mitochondrial short chain enoyl-CoA hydratase was observed in a human glioblastoma cell line persistently infected with measles virus. Knockdown of this gene was found to impair measles virus replication and significantly reduce the cytopathic effects of the virus [23]. This finding suggests a possible interaction between virus replication and lipid metabolism in host cells, and might provide a new strategy for virus control.

#### Anti-oxidant related proteins

We observed a downregulation of malic enzyme and protein disulfide isomerase (PDI) at both 3DPI and 5DPI (Table 1). Malic enzyme catalyzes the interconversion of L-malate and oxaloacetate, with nicotinamide adenine dinucleotide (NAD) as a coenzyme. This reaction produces reduced nicotinamide adenine dinucleotide phosphate (NADPH), which is crucial to cellular anti-oxidative defence strategies in most organisms. PDI is a multifunctional protein that catalyzes thiol–disulfide interchanges underlying the formation, reduction, and rearrangement of secreted and cell-surface-associated proteins [24]. PDI has been demonstrated to play a role in redox control at the cell surface [25]. In response to increased extracellular reduction, PDI may help to re-establish redox homeostasis by forming and rearranging disulfide bonds [26]. PDI is an essential component of the endoplasmic reticulum (ER), which is involved in viral translation, replication, and encapsidation. In particular, PDI has been located in the complex I [27], the main ribonucleoprotein complex formed with the 3'UTR in dengue 4 virus replication. It is therefore likely that PDI plays a role in viral replication, translation, or encapsidation, and modulation of the expression of this protein would interfere with viral replication.

#### Proteins involved in the cell cytoskeleton

Microtubules are components of the cell cytoskeleton that play a central role in cellular trafficking. Here we show that the level of beta-1 tubulin is down-regulated in CHIKV-infected SG at 5DPI (Table 1). Rotavirus infection has been shown to lead to a remodelling of the microtubule network, together with the formation of tubulin granules [28]. Dengue virus has also been demonstrated to interact with tubulin to ensure its entry into mosquito cells [29]. Microtubule-dependent vesicular transport to the ER is a step that is required for infectivity of alphaviruses like Sindbis virus [30]. These observations suggest that cytoskeletal modulations occur after infection by CHIKV and that these may explain in part the pathological changes in mosquito cells observed upon arbovirus infection [31].

### Serine protease inhibitors

Serine proteinase inhibitors (serpins) display conformational polymorphism, shifting from native to cleaved, latent, delta, or polymorphic forms. Many serpins, such as antitrypsin and antichymotrypsin, function as serine protease inhibitors that regulate blood coagulation cascades. In arthropods, they regulate melanization, which plays an important role in immune defence and wound healing. In the *Ae. aegypti* mosquito, two distinct melanization activation pathways were found to be carried out by different modules of serine proteases and their specific serpins. Immune melanization proteases (IMP-1 and IMP-2) and SERPIN-1 mediate hemolymph prophenoloxidase cleavage and the immune response against the malaria parasite [32]. In *Ae. aegypti* salivary glands infected by CHIKV, a high molecular form of SERPIN1 (47 kDa) was found to be down-regulated while a low molecular form (41 kDa) was up-regulated (Table 1). We propose that the higher molecular form may be the inactive precursor of SERPIN1, which is activated by cleavage into the 41kDa form. We found that a putative serpin (gi|18568304) with a molecular weight of 47 kDa was either down-regulated or up-regulated in infected salivary glands. This latter serpin was also found to be up-regulated in DENV-infected *Ae. aegypti* salivary glands [10]. This serpin is distinct from the anti-factor Xa clotting inhibitor. The corresponding differentially modulated spots show the same molecular weights on the gels but possess various pI values, suggesting that posttranslational modifications occur and that the corresponding proteins are differentially modulated during CHIKV infection.

### Immunity-related proteins

As shown in Table 1, an up-regulation of the angiopoietin-like protein variant (gi|94468352) was observed in 3DPI CHIKV-infected salivary glands. An increase of gene expression was also observed in blood-fed *Ae. aegypti* salivary glands [33].

### Proteins involved in blood-feeding

The apyrase gene of the *Ae. aegypti* vector mosquito is expressed specifically in the adult female salivary glands [34]. We found that apyrase was up-regulated in CHIKV-infected salivary glands at both 3 and 5DPI. An up-regulation of the apyrase transcript was also found in DENV-infected salivary glands of the same vector [10]. This enzyme inhibits ADP-dependent platelet aggregation and is therefore essential to perform a blood meal. The level of apyrase influences the probing time of *Anopheles gambiae*[35]. Interestingly, a down-regulation of apyrase was observed in *A. gambiae* infected with *Plasmodium berghei*[7]. An increase of the expression of this protein may facilitate hematophagy in arbovirus-infected mosquitoes.

A 30 kDa protein is abundantly expressed in adult female mosquito salivary glands, which belongs to a family of proteins that appears to be involved in allergic reactions to mosquito saliva [36]. Recently, a member of this family was found in *Ae. aegypti* to be a specific inhibitor of collagen-induced platelet aggregation [37,38]. Interestingly, two mature forms of the protein were identified: a 21.4 kDa form and a 25 kDa one [22]. Two additional splice variants were also identified [39]. Surprisingly, in our study we observed a down-regulation of the 30 kDa protein in 3 DPI salivary glands (gi|2114497) whereas an up-regulation was found at 5DPI. Moreover, a second spot was up-regulated at 5DPI, corresponding to a lower molecular weight form of the protein (gi|108873586). These results suggest both time-dependent and isoform-specific regulation of the 30 kDa protein.

### Antifreeze protein

We found that the level of an antifreeze protein (gi:157103422) was increased in CHIKV-infected SG. Arthropods have evolved physiological and behavioral strategies to survive to low environmental temperatures that are collectively termed “freeze avoidance” or “freeze tolerance.” These processes elicit different adaptive responses, and specific antifreeze proteins are involved [40,41]. Antifreeze proteins are a diverse class of compounds that bind to ice crystals and restrict their growth [42]. They require structural complementarity with ice to adsorb to its surface; however, the molecular mechanisms of blocking ice remain to be elucidated [43]. A recent study showed that the presence of *Anaplasma phagocytophillum* in *Ixodes* ticks increases the level of antifreeze glycoproteins and thereby the resistance of the arthropod to temperature modulation [44]. CHIKV-infected mosquitoes might therefore present a higher resistance to temperature modulations than non-infected mosquitoes.

### Other proteins

Venom antigen (gi|18568284) is up-regulated at 5DPI (Table 1). This is a member of the antigen 5-related family of proteins that are found in the salivary glands of many blood-sucking insects. Three members of this gene family were found in *Ae. aegypti*[39]. They are overrepresented in the sialotranscriptome of this mosquito, indicating that they may be preferentially expressed in the salivary glands. gi|18568284 was found to be exclusively transcribed in female glands, suggesting an anti-hemostatic function of the corresponding protein.

The 34 and 62 kDa gene families are abundantly expressed in adult female salivary glands. They are specific to the *Aedes* family and have been putatively implied in adhesion phenomena as suggested by a PSI-BLAST analysis that reveals cytoskeletal proteins such as actin and myosin [39].

The D7 salivary family of proteins is abundantly expressed in blood-feeding Diptera, and is distantly related to the odorant-binding protein superfamily. In mosquitoes, two subfamilies exist, the long and short D7 proteins. Four of the five short D7 proteins and the D7 long form of *A. gambiae* were found to bind serotonin with high affinity, as well as histamine and norepinephrine [45]. An increase of expression of a short D7 form was observed at both 3DPI and 5DPI. Since we can expect that this protein might possess the same function as the short *A. gambiae* D7 forms, an increase in its expression might favour the vector by decreasing the local reaction and allowing the mosquito to bite without being detected. This is particularly important since a modification of the feeding behaviour was demonstrated for *Aedes triseriatus* infected by La Crosse virus [46]. Infected females made multiple probings to obtain a partial blood meal. They were thereby more exposed to the response of bitten vertebrates.

## Conclusions

CHIKV triggers the modulation of both intracellular and secreted proteins in *Ae. aegypti* salivary glands. Overall, most regulated proteins are over-expressed upon infection.

In agreement with IFA profiles and viral RNA measurements, which both showed that the invasion of salivary glands is not complete at 3DPI, fewer proteins were modulated at that time compared with 5DPI, the time at which equilibrium has been reached in terms of RNA replication, and the three lobes of the salivary glands have been invaded. Interestingly, some modulations were, however, observed as early at 3DPI and some of the proteins identified were only modulated at that time and are therefore early markers of infection. These particular proteins are implicated in cell signalling, fatty acid metabolism and immunity.

Among the modulated salivary gland proteins, most are in favour of the virus and its transmission, either through direct interaction or by acting on blood-feeding success. Only a few were implicated in vector protection. Anti-oxidant proteins were down-regulated whereas the antifreeze protein was up-regulated and this may contribute to the protection of the vector against climatic modulations. Several CHIKV up-regulated proteins such as beta-1 tubulin and the 34 and 62 kDa proteins had an effect on virus transport. Other proteins are probably implied in blood-feeding success, like a short D7 form, adenosine deaminase, and inosine-uridine preferring nucleoside hydrolase, which may exert an anti-infammatory effect at the bite site. Among the up-regulated proteins, several might be interesting candidates in the design of markers of exposure to non-infected and infected bites as suggested by the results in the case of dengue transmission by *Ae. aegypti*[47].

## Abbreviations

*Ae. aegypti*: *Aedes aegypti*; *A. gambiae*: *Anopheles gambiae*; CHIKV: Chikungunya virus; 2DE-MS/MS: Two dimensional electrophoresis coupled to tandem mass spectrometry; PFU: Plaque forming unit; SGE: Salivary gland extract; SG: Salivary gland; IFA: Indirect immunofluorescence assay; RT-qPCR: Reverse transcription quantitative polymerase chain reaction; DPI: Day post-infection; MALDI-TOF/TOF: Matrix-Assisted Laser Desorption/Ionisation with time-of-flight mass spectrometry; ADA: Adenosine deaminase; PDI: Protein disulfide isomerase.

## Competing interests

The authors declare that they have no competing interests.

## Authors’ contributions

ST-N and EB prepared and developed the 2DE gels and analyzed the profiles using dedicated software. PL, J-CR and AN carried out the mass spectrometry analyses. VC designed the experiments, performed artificial infections in BSL3, dissected salivary glands, analyzed the data and wrote the manuscript. All authors read and approved the final version of the manuscript.

## Supplementary Material

Additional file 1 **Figure S1. **Salivary gland extract profiles of control, 3DPI, 5DPI infected mosquitoes. 130 μg of salivary gland extract from 3 DPI (A) and 5 DPI (B) CHIKV-infected mosquitoes and control mosquitoes (C) were loaded onto 3–10 NL immobilins. The immobilins were then deposited on the top of 12% SDS-PAGE gels. Spots were revealed using SYPRO Ruby. Click here for file

Additional file 2 **Figure S2. **Spots down-regulated at 3DPI in *Ae. aegypti *salivary glands infected by CHIKV. Gel profiles were compared using Image Master Platinum software. The spots that were found down-regulated at 3DPI are indicated by circles (fold change>1.8; Anova<0.05). Click here for file

Additional file 3 **Figure S3. **Spots down-regulated at 5 DPI in *Ae. aegypti *salivary glands infected by CHIKV. Gel profiles were compared using Image Master Platinum software. The spots that were found down-regulated at 5DPI are indicated by circles (fold change>1.8; Anova<0.05). Click here for file

Additional file 4 ** Table S1. **List of proteins down-regulated at 3DPI in *Ae. aegypti *salivary glands infected with CHIKV identified by mass spectrometry. Click here for file

Additional file 5 **Table S2. **List of proteins up-regulated at 3DPI in *Ae. aegypti *salivary glands infected with CHIKV identified by mass spectrometry. Click here for file

Additional file 6 **Table S3. **List of proteins down-regulated at 5DPI in *Ae. aegypti *salivary glands infected with CHIKV identified by mass spectrometry. Click here for file

Additional file 7 **Table S4. **List of proteins up-regulated at 5DPI in *Ae. aegypti *salivary glands infected with CHIKV identified by mass spectrometry. Click here for file
