# Supplementary figures and images for: Infection by chikungunya virus modulates the expression of several proteins in Aedes aegypti salivary glands
Source: Parasit Vectors. 2012 Nov 15;5:264. doi: 10.1186/1756-3305-5-264 (PMC3549772; doi:10.1186/1756-3305-5-264)

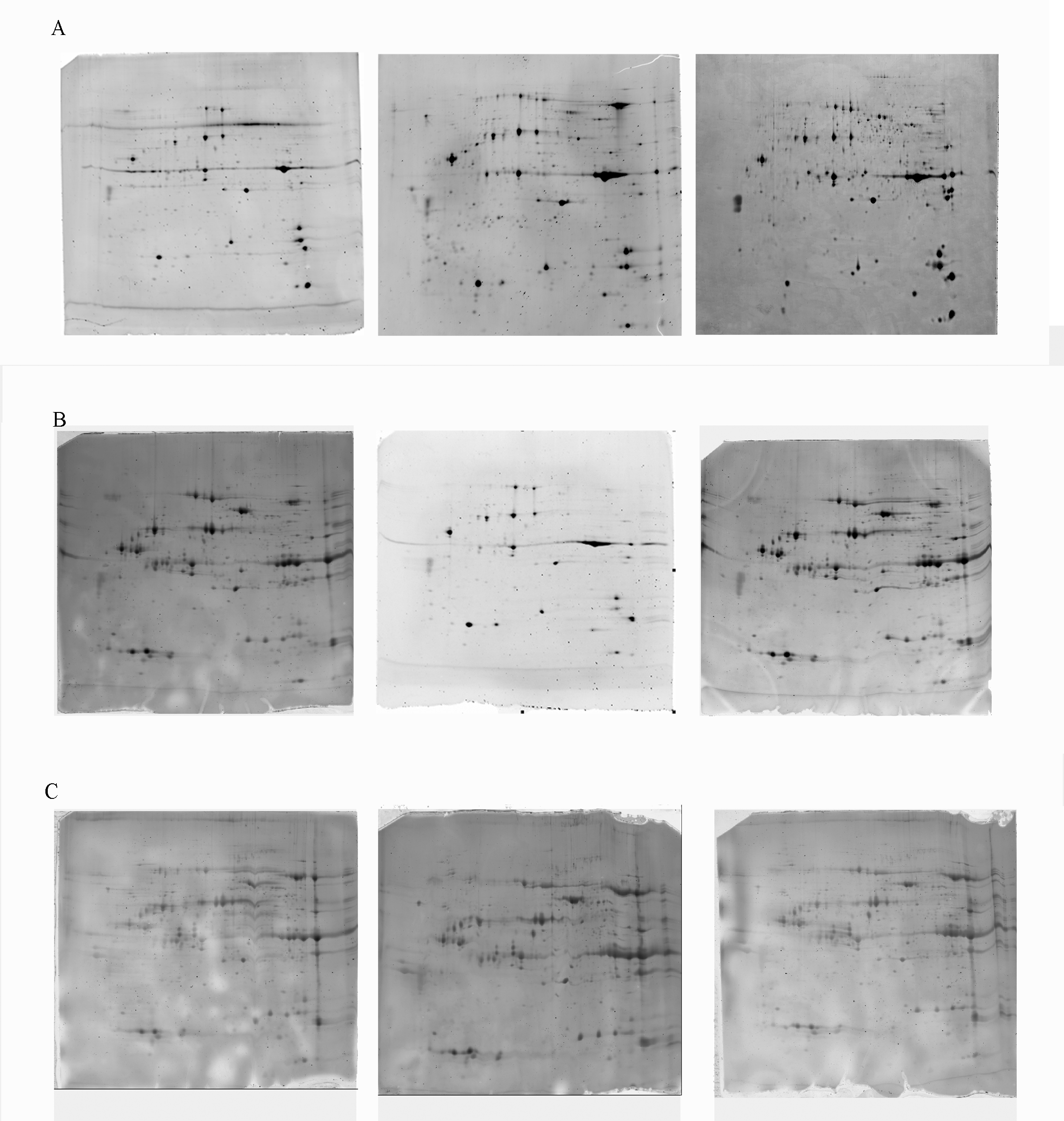

Supplement: Additional file 1 — Figure S1. Salivary gland extract profiles of control, 3DPI, 5DPI infected mosquitoes. 130 μg of salivary gland extract from 3 DPI (A) and 5 DPI (B) CHIKV-infected mosquitoes and control mosquitoes (C) were loaded onto 3–10 NL immobilins. The immobilins were then deposited on the top of 12% SDS-PAGE gels. Spots were revealed using SYPRO Ruby. [file 1756-3305-5-264-S1.tiff]

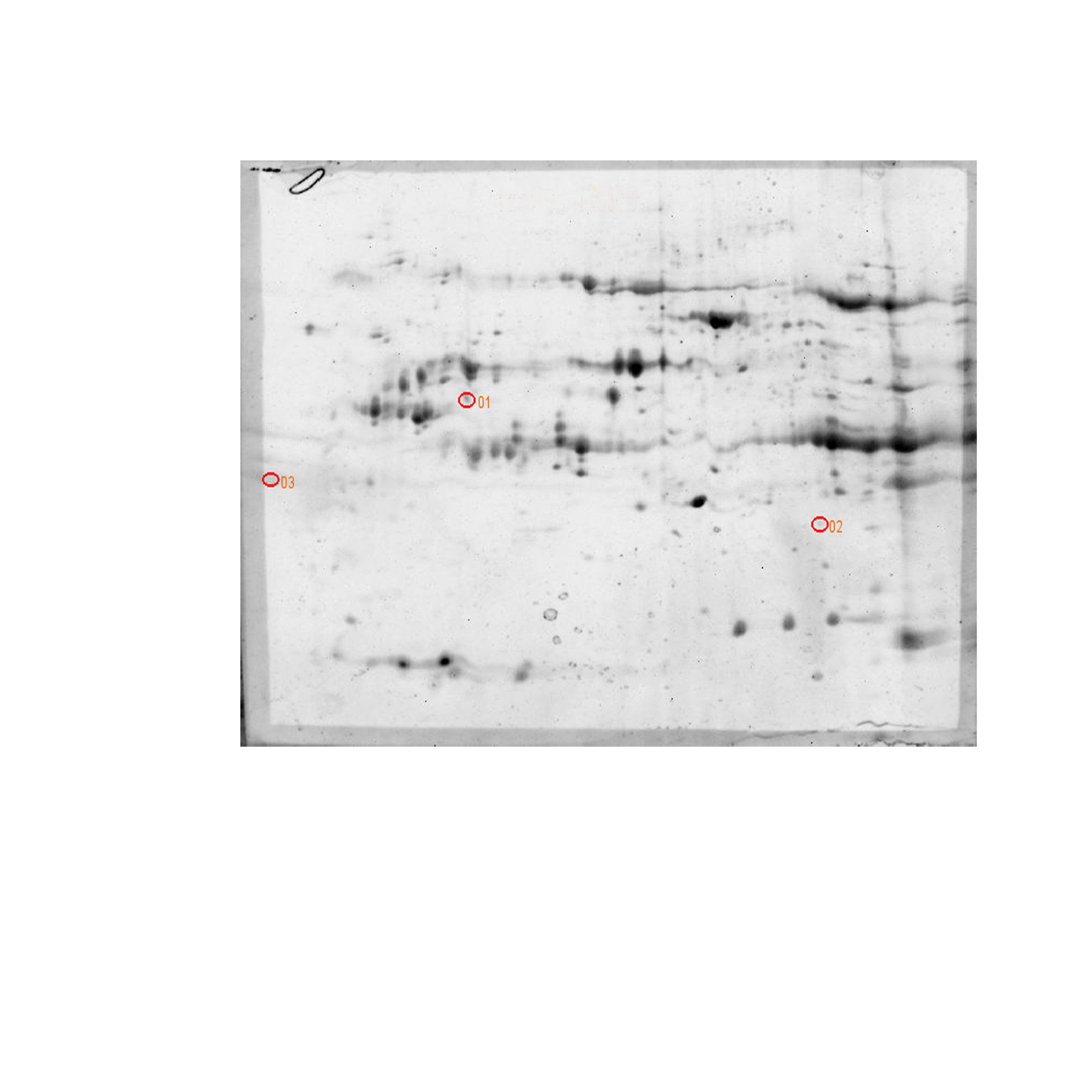

Supplement: Additional file 2 — Figure S2. Spots down-regulated at 3DPI in Ae. aegypti salivary glands infected by CHIKV. Gel profiles were compared using Image Master Platinum software. The spots that were found down-regulated at 3DPI are indicated by circles (fold change>1.8; Anova<0.05). [file 1756-3305-5-264-S2.tiff]

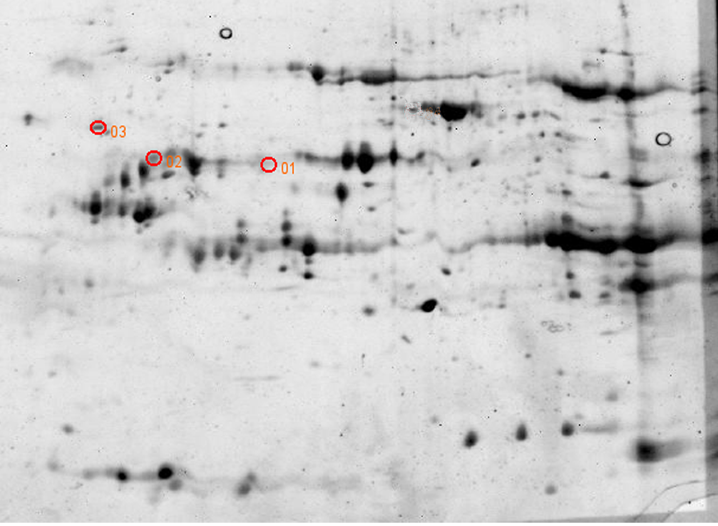

Supplement: Additional file 3 — Figure S3. Spots down-regulated at 5 DPI in Ae. aegypti salivary glands infected by CHIKV. Gel profiles were compared using Image Master Platinum software. The spots that were found down-regulated at 5DPI are indicated by circles (fold change>1.8; Anova<0.05). [file 1756-3305-5-264-S3.tiff]
