# Supplementary material for: Infection by chikungunya virus modulates the expression of several proteins in Aedes aegypti salivary glands
Source: Parasit Vectors. 2012 Nov 15;5:264. doi: 10.1186/1756-3305-5-264 (PMC3549772; doi:10.1186/1756-3305-5-264)
Supplement: Additional file 4 — Table S1. List of proteins down-regulated at 3DPI in Ae. aegypti salivary glands infected with CHIKV identified by mass spectrometry. [file 1756-3305-5-264-S4.doc]

Supplementary Table 1: Proteins down-regulated in CHIKV-infected females at d3 post-infection

| Genebank and Vector base Identification | Protein  Family/Description | Predicted  Mr | Spot number | Peptide  count | Protein  score | MS + MS/MS  Peptide sequence | Comments | Subcellular  Localization | Anova | Fold |
| --- | --- | --- | --- | --- | --- | --- | --- | --- | --- | --- |
| gi|108883990/  AAEL000726-PA | fibrinogen and fibronectin [Aedes aegypti] | 33896.5 | 1 | 7 | 312 | | MMMRLK | | --- | | MMMRLK | | MMMRLK | | MMMRLK | | MMMRLK | | GGWWYYR | | GGWWYYR | | IKHLLQEK | | IKHLLQEK | | SISWVWFSTEK | | YAGYDAFAVGPEEER | | YAGYDAFAVGPEEER | | YAGYDAFAVGPEEERYP | | LAK | | YAGYDAFAVGPEEERYP | | LAK | | Intracellular cell signalling processes  Fibrinogen is involved in blood clotting | secreted | 0.03 | 6 |
| gi|108874624  gi|157121273  AAEL009287-PA | ran | 24681.6 | 2 | 6 | 183 | | AIVFHRK | | --- | | AIVFHRK | | NVPNWHR | | NVPNWHR | | HMTGEFEKK | | HMTGEFEKK | | NLQYYDISAK | | NLQYYDISAK | | FNVWDTAGQEK | | FNVWDTAGQEK | | MDKDWQQQIEK | | Ras GTPases domain involved in intracellular cell signalling processes  Ran GTPases domain involved in nucleocytoplasmic transport | intracellular | 0.049 | Not detected in non infected |
| gi|2114497  AAEL010235-PA  e) | 30 kDa salivary gland allergen Aed a 3 [Aedes aegypti] | 27414.6 | 3 | 12 | 748 | | QVVALLDK | | --- | | SCVSSKGR | | VPVVEAIGR | | VPVVEAIGR | | NDPADTYR | | NDPADTYR | | VISEEEKK | | QVVALLDKDTK | | QVVALLDKDTK | | QVVALLDKDTK | | VDHIQSEYLR | | VDHIQSEYLR | | SALNNDLQSEVR | | SALNNDLQSEVR | | GSEKNDPADTYR | | GSEKNDPADTYR | | SEYQCSEDSFAAAK | | SEYQCSEDSFAAAK | | KSEYQCSEDSFAAAK | | KSEYQCSEDSFAAAK | | SALNNDLQSEVRVPVVE | | AIGR | | unknown | secreted | 0.006 | -3 |
