# Supplementary material for: Infection by chikungunya virus modulates the expression of several proteins in Aedes aegypti salivary glands
Source: Parasit Vectors. 2012 Nov 15;5:264. doi: 10.1186/1756-3305-5-264 (PMC3549772; doi:10.1186/1756-3305-5-264)
Supplement: Additional file 5 — Table S2. List of proteins up-regulated at 3DPI in Ae. aegypti salivary glands infected with CHIKV identified by mass spectrometry. [file 1756-3305-5-264-S5.doc]

Supplementary Table 2: Proteins upregulated in salivary gland extracts of *Aedes aegypti* chik-infected females at D3 post-infection

| Genebank and Vector base Identification | Protein  Family/Description | Predicted  Mr | Spot  number | Peptide  count | Protein  score | MS + MS/MS  Peptide sequence | Comments | Subcellular  localization | Anova | Fold  change |
| --- | --- | --- | --- | --- | --- | --- | --- | --- | --- | --- |
| gi|157113141/  AAEL006347 | apyrase, putative [Aedes aegypti] | 63170.9 | 1, 2 | 17 | 1 100 | | DGFSAMKR | | --- | | VQQDPQILK | | VQQDPQILK | | TGPLDSDVFK | | IVIDISKPVR | | IVIDISKPVR | | TNCLQVSGLR | | TNCLQVSGLR | | IEVMDYTNPK | | PFAIIQAGNFR | | RTGPLDSDVFK | | RTGPLDSDVFK | | KIEVMDYTNPK | | KIEVMDYTNPK | | KIEVMDYTNPK | | NWEGYPVFIDHK | | NWEGYPVFIDHK | | EAEYYIVVPSYLADGK | | EAEYYIVVPSYLADGK | | VQQDPQILKDLVPWR | | ITNGDIIEAAPFGSTADLIR | | ITNGDIIEAAPFGSTADLIR | | GADIWDVAEHSFALDDE | | GR | | GADIWDVAEHSFALDDE | | GR | | LKGADIWDVAEHSFALDD | | EGR | | LKGADIWDVAEHSFALDD | | EGR | | VEAIGSTVVGETMIELDR | | DSCR | | LFPLTLIHINDLHARFEET | | NMK | | Anti-platelet | secreted | 4.9910-4/0.00 | 3.2/3.4 |
| gi|18568296  AAEL003601 | putative 34 kDa secreted protein [Aedes aegypti] | 36382.,3 | 3, 15 | 14 | 712 | | IYLGALR | | --- | | IYLGALR | | SRDIFEK | | SRDIFEK | | VFDGILKR | | VFDGILKR | | EHGEMLER | | EHGEMLER | | ERQVDLYR | | ERQVDLYR | | ERQVDLYR | | SVATEIVQMR | | SVATEIVQMR | | SVATEIVQMR | | VTELEQQIAK | | VTELEQQIAK | | DSAISTDQVDQLK | | DSAISTDQVDQLK | | QNFEDQVNQIVK | | QNFEDQVNQIVK | | QNFEDQVNQIVK | | FLNHMNDQELIGK | | FLNHMNDQELIGK | | LEELMNKLETNYR | | HMMEEKLEELMNK | | QSGTQATKEHGEMLER | | QTASMYEDMAELIFQR | | QTASMYEDMAELIFQR | |  | secreted | 0.0023 | 2.1/7 |
| gi|18568330  gi|61742025  **)** | putative 16.9 kDa secreted protein [Aedes aegypti]  putative 14.5 kDa secreted protein [Aedes aegypti] | 17384.3 | 5 | 8 | 562 | | TELDEVTK | | --- | | NLQVDGNMPK | | NLQVDGNMPK | | NLQVDGNMPK | | QLSDDKPLQCK | | QLSDDKPLQCK | | QLSDDKPLQCK | | QLSDDKPLQCK | | EADFFKQNLCN | | EADFFKQNLCN | | IRNLQVDGNMPK | | IRNLQVDGNMPK | | VAQDMVPYGFNIK | | VAQDMVPYGFNIK | | VAQDMVPYGFNIK | | VAQDMVPYGFNIK | | EYMNCAFESSGWAK | | EYMNCAFESSGWAK | | VKEYMNCAFESSGWAK | | VKEYMNCAFESSGWAK | | VKEYMNCAFESSGWAK | |  | secreted | 0.005 | 1.9 |
| gi|157115994  AAEL007394 | hypothetical protein  2 | 18953,8 | 6 | 10 | 299 | | EVEFFSK | | --- | | EVEFFSK | | MLCMAYR | | MLCMAYR | | MLCMAYR | | MLCMAYR | | LGPMSYYR | | LGPMSYYR | | LGPMSYYR | | LGPMSYYR | | VGWMDKGTR | | NREVEFFSK | | NREVEFFSK | | QKMLCMAYR | | NKLGPMSYYR | | NKLGPMSYYR | | QGPGEFKQMLR | | QGPGEFKQMLR | | QGPGEFKQMLR | | SNSMYAFMDCTFIR | | SNSMYAFMDCTFIR | | SNSMYAFMDCTFIR | | TAELTEIEAMCNMEFR | | TAELTEIEAMCNMEFR | |  | | TAELTEIEAMCNMEFR | | Short salivary D7 form | secreted | 0.0065 | 1.9 |
| gi|157113519 | inosine-uridine preferring nucleoside hydrolase [Aedes | 38196.3 | 7, 18 | 9 | 685 | | MHELIR | | --- | | MHELIR | | ILEGLGRR | | ILEGLGRR | | RDVPLYR | | RDVPLYR | | NSVFKEPK | | NSVFKEPK | | YKADVELAGK | | YKADVELAGK | | SEIAGIYILGGNR | | SEIAGIYILGGNR | | GMMAILYHDNDEK | | GMMAILYHDNDEK | | GMMAILYHDNDEK | | YNVVVEAITCTHGNTDLE | | NSVTNAAR | | YNVVVEAITCTHGNTDLE | | NSVTNAAR | | KYNVVVEAITCTHGNTDL | | ENSVTNAAR | | KYNVVVEAITCTHGNTDL | | ENSVTNAAR | | Hydrolase activity  Not present in male saliva  Maybe involved in blood feeding | intracellular | 0.0083 | 2.1/4.6 |
| gi|1094353 | apyrase | 63146.9 | 8, 16 | 18 | 1110 | | DGFSAMKR | | --- | | SDELKPLDK | | VQQDPQILK | | VQQDPQILK | | TGPLDSDVFK | | TGPLDSDVFK | | TNCLQVSGLR | | TNCLQVSGLR | | IVIDISKPIR | | IVIDISKPIR | | IELDRDSCR | | IELDRDSCR | | IEVMDYTNPK | | PFAIIQAGNFR | | RTGPLDSDVFK | | RTGPLDSDVFK | | VEAIGSTVVGETK | | KIEVMDYTNPK | | KIEVMDYTNPK | | KIEVMDYTNPK | | EKVEAIGSTVVGETK | | EKVEAIGSTVVGETK | | QAEYYIVVPSYLADGK | | QAEYYIVVPSYLADGK | | VQQDPQILKDLVPWR | | ITNGDIIEAAPFGSTADLIR | | ITNGDIIEAAPFGSTADLIR | | GADIWDVAEHSFALDDE | | GR | | GADIWDVAEHSFALDDE | | GR | | LKGADIWDVAEHSFALDD | | EGR | | Anti-platelet | secreted | 0.012/0.024 | 3.8 |
| gi|157103422 | antifreeze protein, putative [Aedes aegypti] | 18055.7 | 9 | 9 | 598 | | RFVCEQA | | --- | | LAVLDSEQK | | QQQVEELAQR | | QQQVEELAQR | | QQQVEELAQR | | QQQVEELAQR | | VELWIGASDLAR | | VELWIGASDLAR | | TAKVELWIGASDLAR | | FMWHPTGLDVSYSK | | FMWHPTGLDVSYSK | | FMWHPTGLDVSYSK | | LINWHWNDVVCASMR | | LINWHWNDVVCASMR | | DGYEHCVHLWYEPSR | | DGYEHCVHLWYEPSR | | WIAGMPDNKDGYEHCVH | | LWYEPSR | | WIAGMPDNKDGYEHCVH | | LWYEPSR | | bind to ice and inhibit its growth in a non colligative manner | secreted | 0.008 | 2.2 |
| gi|157115994  AAEL007394 | hypothetical protein | 18953.8 | 10 | 11 | 309 | | EVEFFSK | | --- | | FNFYGYK | | FNFYGYK | | MLCMAYR | | MLCMAYR | | MLCMAYR | | LGPMSYYR | | LGPMSYYR | | LGPMSYYR | | LGPMSYYR | | VGWMDKGTR | | QKMLCMAYR | | NKLGPMSYYR | | NKLGPMSYYR | | QGPGEFKQMLR | | QGPGEFKQMLR | | QGPGEFKQMLR | | MAADMHASGFPDR | | MAADMHASGFPDR | | MAADMHASGFPDR | | SNSMYAFMDCTFIR | | SNSMYAFMDCTFIR | | SNSMYAFMDCTFIR | | SNSMYAFMDCTFIR | | TAELTEIEAMCNMEFR | | TAELTEIEAMCNMEFR | |  | | TAELTEIEAMCNMEFR | | Insect pheromone/odorant-binding proteins domain | secreted | 0.025 | 1.9 |
| gi|18568304 | putative serpin | 47315.1 | 11 | 19 | 1070 | | QISVETR | | --- | | QISVETR | | GFQGENVK | | VFMSLYK | | HTLPVDEK | | LWLIMPDR | | LWLIMPDR | | LWLIMPDR | | LWLIMPDR | | QIENGLTAQK | | PIGTSFSDKR | | MSEIEAFANK | | MSEIEAFANK | | MSEIEAFANK | | FLEGSISAGYAK | | FLEGSISAGYAK | | LVDGKDDLYVIK | | DFNDQLSVESIR | | DFNDQLSVESIR | | FDRPFVMMMLSK | | FDRPFVMMMLSK | | FDRPFVMMMLSK | | FDRPFVMMMLSK | | FDRPFVMMMLSK | | FDRPFVMMMLSK | | FDRPFVMMMLSK | | VEVVELISDKVDGVK | | FVEGQNLVVAPLLTFR | | FVEGQNLVVAPLLTFR | | EGNVPILLANYFSPTDK | | EGNVPILLANYFSPTDK | | ASSIKDFNDQLSVESIR | | ASSIKDFNDQLSVESIR | | FDLHSLLGIQQDTSVEK | | RFVEGQNLVVAPLLTFR | | EGNVPILLANYFSPTDKL | | R | | EGNVPILLANYFSPTDKL | | R | | unlikely to be inhibitory | secreted | 0.036 | 2 |
| gi|157104013 | cyclohex-1-ene-1-carboxyl-CoA hydratase, putative | 31909.1 | 14 | 6 | 123 | | HFHATFSTK | | --- | | RHFHATFSTK | | NVALITLNRPK | | NVALITLNRPK | | DRLEGMTAFVEK | | FGQPEIALGTIPGAGGSQ | | R | | FGQPEIALGTIPGAGGSQ | | R | | AFAAGADIKEMQNNTYA | | K | | [enoyl-CoA hydratase/isomerase family](http://www.uniprot.org/uniprot/?query=family:"enoyl-CoA+hydratase%2Fisomerase+family")  catalytic activity  plays a particularly important role in the [metabolism](http://en.wikipedia.org/wiki/Metabolism) of [unsaturated fatty acids](http://en.wikipedia.org/wiki/Unsaturated_fatty_acid) | secreted | 0.026 | 4 |
| gi|94468352 | angiopoietin-like protein variant [Aedes aegypti] | 33515.4 | 15 | 14 | 542 | | TWAEYR | | --- | | TWAEYR | | YAWYDK | | VPALYER | | VPALYER | | FVVGPEEQR | | FVVGPEEQR | | YDGSVKFDR | | YDGSVKFDR | | GGWWYYSGK | | GGWWYYSGK | | FSDVSNTPLK | | CYGSSLTGIWK | | YGGDWLVVMHR | | YGGDWLVVMHR | | SSGLTTIPIGSEPR | | SSGLTTIPIGSEPR | | ERYGGDWLVVMHR | | GSGFSTYDNDDFGCSNK | | GSGFSTYDNDDFGCSNK | | YAWYDKFVVGPEEQR | | SYELMVEMEDFNGNLK | | Immune related protein | secreted | 0.015 | 4.5 |
| gi|108883988  AAEL000732 | conserved hypothetical protein | 65165.2 | 13, 17 | 28 | 1520 | | YGFMGLK | | --- | | VEEYQR | | YGFMGLK | | YGFMGLK | | TQIEGLLK | | FLEEQKR | | GIKQDFEK | | DKLGDFYR | | DTIEWNLK | | RTQIEGLLK | | TEVDHLLVR | | IESQFSDYK | | IESQFSDYK | | STIVFSTWTK | | STIVFSTWTK | | KTEVDHLLVR | | KTEVDHLLVR | | GYVKVEEYQR | | LTVVCNNYYK | | LTVVCNNYYK | | FDPHVPDEIPK | | FDPHVPDEIPK | | VVVADYGCTSFR | | VVVADYGCTSFR | | DINHLPHVLVAR | | DINHLPHVLVAR | | KLDEEVGTLHEK | | NALDHMFVSQMK | | NALDHMFVSQMK | | NALDHMFVSQMK | | NALDHMFVSQMK | | NALDHMFVSQMK | | CVVATGNDFDVHK | | NSDAKDTIEWNLK | | NSDAKDTIEWNLK | | VFGALVGDTSDFGSIR | | VFGALVGDTSDFGSIR | | DLCVTYLNSNQMSK | | DLCVTYLNSNQMSK | | DLCVTYLNSNQMSK | | CVVATGNDFDVHKDK | | CVVATGNDFDVHKDK | | RLEGEDSVLLEATVLK | | RLEGEDSVLLEATVLK | | TLNDELVELQQHTSEK | | TLNDELVELQQHTSEK | | LFEATLSSLPNSLTELR | | IVTPSGNPMSNVNLHLEG | | NSLHNYVATPK | | IVTPSGNPMSNVNLHLEG | | NSLHNYVATPK | |  | secreted | 0.034/0.031 | 2.2/4 |
