# Supplementary material for: Infection by chikungunya virus modulates the expression of several proteins in Aedes aegypti salivary glands
Source: Parasit Vectors. 2012 Nov 15;5:264. doi: 10.1186/1756-3305-5-264 (PMC3549772; doi:10.1186/1756-3305-5-264)
Supplement: Additional file 6 — Table S3. List of proteins down-regulated at 5DPI in Ae. aegypti salivary glands infected with CHIKV identified by mass spectrometry. [file 1756-3305-5-264-S6.doc]

Supplementary table 3: Proteins downregulated in salivary gland extracts of *Aedes aegypti* Chik-infected females at J5 postinfection

| Genebank and Vector base Identification | Protein  Family/Description | Predicted  Mr | Spot number | Peptide  count | Protein  score | MS + MS/MS  Peptide sequence | Comments | Subcellular  localization | Anova | Fold change |
| --- | --- | --- | --- | --- | --- | --- | --- | --- | --- | --- |
| gi|157135059  AAEL003182 | SERPIN1 protein precursor, putative | 47237.1 | 1,2 | 12 | 394 | | QISVETR | | --- | | GFQGENVK | | VFMSLYK | | LWLIMPDR | | LWLIMPDR | | PIGTSFSDKR | | MSEIEAFANK | | FLEGSISAGYAK | | FLEGSISAGYAK | | LVDGKDDLYVIK | | DFNDQLSVESIR | | DFNDQLSVESIR | | FDRPFVMMMLSK | | FDRPFVMMMLSK | | FVEGQNLVVAPLLTFR | | ASSIKDFNDQLSVESIR | | ASSIKDFNDQLSVESIR | | Unlikely to be inhibitory | secreted | 0.04 | 10 |
| gi|157107430 | protein disulfide isomerase | 27414.6 | 3 | 8 | 214 | | ILEFFGMK | | --- | | ILEFFGMK | | PETNDLAADK | | ILEFFGMKK | | NGTPIEYTGGR | | NGTPIEYTGGR | | FADHESIVIAK | | INSFPTIYLYR | | INSFPTIYLYR | | AVFDGEYTEEALKK | | ILFVTIDADQEDHQR | | ILFVTIDADQEDHQR | | Regulates the activity of target proteins through changes in the redox state of thiol groups | secreted | 0.03 | 2 |
| gi|111035024 | beta-1 tubulin | 48452.2 | 3 | 7 | 90 | | TAVCDIPPR | | --- | | YLTVAAVFR | | YLTVAAVFR | | FPGQLNADLR | | FPGQLNADLR | | LAVNMVPFPR | | ISEQFTAMFR | | ISEQFTAMFR | | ISEQFTAMFR | | FPGQLNADLRK | | INVYYNEASGGK | | Cytoskeleton protein | intracellular | 0.03 | 2 |
| gi|108883625 | malic enzyme [Aedes aegypti] | 72552.2 | 4 | 17 | 407 | | FLDKYR | | --- | | AIVVTDGER | | GYVYEVLR | | GYVYEVLR | | GLFVTINDR | | GLFVTINDR | | GLAFTLEER | | NWPEHDVR | | NWPEHDVR | | WPPQQEAPK | | GSLYPPLSAIR | | GSLYPPLSAIR | | QTLGIHGLQPAR | | QTLGIHGLQPAR | | QTLGIHGLQPAR | | YLYLVDLQDR | | GLASTYPEPQDK | | CIFSSGSPFPPVK | | AMQAEGCGLQEAR | | FKSQEEQLELCR | | FKSQEEQLELCR | | YLYLVDLQDRNEK | | YLYLVDLQDRNEK | | AECTAQAAYENTEGR | | NNARPIIFALSNPTSK | | NNARPIIFALSNPTSK | | Lipogenesis  Involved in antioxidant defence  Involved in the tricarboxylic cycle | intracellular | 0.02 | 2.3 |
