# Supplementary material for: Infection by chikungunya virus modulates the expression of several proteins in Aedes aegypti salivary glands
Source: Parasit Vectors. 2012 Nov 15;5:264. doi: 10.1186/1756-3305-5-264 (PMC3549772; doi:10.1186/1756-3305-5-264)
Supplement: Additional file 7 — Table S4. List of proteins up-regulated at 5DPI in Ae. aegypti salivary glands infected with CHIKV identified by mass spectrometry. [file 1756-3305-5-264-S7.doc]

Supplementary table 4: Proteins upregulated in salivary gland extracts of *Aedes aegypti* chik-infected females at D5 post-infection

| Genebank and Vector base Identification | Protein  Family/  Description | Predicted  Mr | Spot number | Peptide  count | Protein  score | MS + MS/MS  Peptide sequence | Comments  role | Subcellular  localization | Anova | Fold change |
| --- | --- | --- | --- | --- | --- | --- | --- | --- | --- | --- |
| gi|108881297 | SERPIN1 protein precursor, putative [Aedes aegypti] | 41460.1 | 1 | 6 | 300 | | SISISVAR | | --- | | TLLEGEESR | | AQYEDIAQK | | YEELQITAGR | | YEELQITAGR | | NMQDMVEDYR | | NMQDMVEDYR | | NSKIEISELNR | | LNDLEDALQQAK | | SLNNQFASFIDK | | TNAENEFVTIKK | | WELLQQVDTSTR | | WELLQQVDTSTR | | SLNNQFASFIDKVR | | SLNNQFASFIDKVR | | SGGGFSSGSAGIINYQR | | FSSCGGGGGSFGAGGG | | FGSR | | GGGGGGYGSGGSSYGS | | GGGSYGSGGGGGGGR | | Immune-related protein | secreted | 0.026 | 2.7 |
| gi|157113519  AAEL006485-PA | inosine-uridine preferring nucleoside hydrolase [Aedes aegypti] | 38196.3 | 5, 16 | 10 | 758 | | MHELIR | | --- | | MHELIR | | ILEGLGRR | | ILEGLGRR | | RDVPLYR | | RDVPLYR | | NSVFKEPK | | NSVFKEPK | | QMVEDLNR | | QMVEDLNR | | QMVEDLNR | | QMVEDLNR | | QMVEDLNR | | QMVEDLNR | | QMVEDLNR | | YKADVELAGK | | YKADVELAGK | | SEIAGIYILGGNR | | SEIAGIYILGGNR | | DFNVNVIDGIEEDTLR | | DFNVNVIDGIEEDTLR | | YNVVVEAITCTHGNTDLE | | NSVTNAAR | | YNVVVEAITCTHGNTDLE | | NSVTNAAR | | KYNVVVEAITCTHGNTDL | | ENSVTNAAR | | KYNVVVEAITCTHGNTDL | | ENSVTNAAR | | Hydrolase activity  Not present in male saliva  Maybe involved in blood feeding | secreted | 0.011 | 1.9/2.4 |
| gi|108880897  AAEL003585  AAEL007872  AAEL003601 | conserved hypothetical protein [Aedes aegypti] | 36068.3 | 7, 18 | 11 | 382 | | IYLGALR | | --- | | IYLGALR | | RVDLYR | | RVDLYR | | SRDIFEK | | VFDGILKR | | VFDGILKR | | SVATEIVQMR | | SVATEIVQMR | | SVATEIVQMR | | VTELEQQIAK | | VTELEQQIAK | | QNFEEQVQQIVK | | FLNHMNDQELIGK | | FLNHMNDQELIGK | | LEELMNKLETNYR | | HMMEEKLEELMNK | | QTASMYEDMAELIFQR | | Belongs to the 34 kDa family | secreted | 0.004 | 2 |
| gi|157113141  AAEL006347 | apyrase, putative [Aedes aegypti] | 63146.9 | 10, 12, 19, 32 | 6/11 | 103/572 | | VQQDPQILK | | --- | | IVIDISKPVR | | IVIDISKPVR | | TNCLQVSGLR | | TNCLQVSGLR | | KIGIIGVLYDK | | KIGIIGVLYDK | | RTGPLDSDVFK | | RTGPLDSDVFK | | KIEVMDYTNPK   | DGFSAMKR | | --- | | DGFSAMKR | | TGPLDSDVFK | | TGPLDSDVFK | | TNCLQVSGLR | | TNCLQVSGLR | | IVIDISKPIR | | IVIDISKPIR | | IELDRDSCR | | IELDRDSCR | | IEVMDYTNPK | | IEVMDYTNPK | | RTGPLDSDVFK | | RTGPLDSDVFK | | VEAIGSTVVGETK | | KIEVMDYTNPK | | KIEVMDYTNPK | | KIEVMDYTNPK | | ITNGDIIEAAPFGSTADLIR | | ITNGDIIEAAPFGSTADLIR | | GADIWDVAEHSFALDDE | | GR | | | Apyrase Precursor (EC 3.6.1.5)(Adenosine diphosphatase)(ATP-diphosphohydrolase)(ATP-diphosphatase)(ADPase)(Allergen Aed a 1)  Anti-platelet | secreted | 4.68 e^-4^ | 1.9/2.2/2.5 |
| gi|108873586  gi|157167432  gi|18568322 | putative 30 kDa allergen-like protein [Aedes aegypti | 27414.6 | 13 | 8 | 346 | | QVVALLDK | | --- | | SCVSSKGR | | VPVVEAIGR | | VPVVEAIGR | | NDPADTYR | | VDHIQSEYLR | | VDHIQSEYLR | | SALNNDLQSEVR | | SALNNDLQSEVR | | SEYQCSEDSFAAAK | | SEYQCSEDSFAAAK | | KSEYQCSEDSFAAAK | |  | secreted | 0.001 | 3 |
| gi|2114497  AAEL010235-PA | 30 kDa salivary gland allergen Aed a 3 [Aedes aegypti | 23797.4 | 14 | 5 | 216 | | SELAADIQR | | --- | | SELAADIQR | | VIEQLDQIK | | VIEQLDQIK | | AIEEDVKGFK | | AIEEDVKGFK | | DSNAYQCSQDR | | VIEQLDQIKVDNVEDGHE | | R | | VIEQLDQIKVDNVEDGHE | | R | |  | secreted | 0.0001 | Not detected in non-infected salivary gland |
| gi|94468642  gi|108880896  gi|157137395  gi|18568296  AAEL003600  AAEL003601 | putative 34 kDa family secreted salivary protein [Aedes | 36092.5 | 20, 23 | 19 | 1020 | | LQLEMAK | | --- | | NKDIFER | | NKDIFER | | MHELIMK | | LVLWEMVK | | LVLWEMVK | | LVLWEMVK | | LVLWEMVK | | TVATDMEVLK | | TVATDMEVLK | | LQEEIEEQTK | | LQEEIEEQTK | | VLNTILDQVNK | | VLNTILDQVNK | | NSLVVVWCWK | | NSLVVVWCWK | | LYKPEEVEIGK | | LYKPEEVEIGK | | QCNLSEDDLTK | | KVLNTILDQVNK | | KVLNTILDQVNK | | TDRLVLWEMVK | | ESFEEKLNDLAK | | TQGVSNMEVQLLR | | TQGVSNMEVQLLR | | TQGVSNMEVQLLR | | TQGVSNMEVQLLR | | FNSETVYGTTDEDQK | | FNSETVYGTTDEDQK | | QMYVDMIEYIFER | | QMYVDMIEYIFER | | QMYVDMIEYIFER | | QANQDTSKAEGEMVEK | | QMYVDMIEYIFER | | QMYVDMIEYIFER | | QANQDTSKAEGEMVEK | | AANEDILPSTTLAACPML | | K | | AANEDILPSTTLAACPML | | K | | AANEDILPSTTLAACPML | | K | | FNSETVYGTTDEDQKSF | | HLAK | |  | secreted | 0.025 | 2.2/2.1 |
| gi|108883987  gi|157109431  gi|18568300  AAEL000748 | conserved hypothetical protein [Aedes aegypti] | 66652.7 | 21, 30 | 14 | 365 | | GIHFQLR | | --- | | GIHFQLR | | FKSNVLGR | | DDYYSLR | | DDYYSLR | | LMNKGYVK | | EIQDLLEK | | GIHFQLRR | | MFLTQMEK | | GYVKAEEFAK | | TASNDCTPFR | | LLVVSTNYYK | | LLVVSTNYYK | | VPHVVVAQYGLK | | VPHVVVAQYGLK | | IFDQWTQDLAK | | IFDQWTQDLAK | | MYDESITALPDSLK | | TELQYDPHVPDEVVR | | TELQYDPHVPDEVVR | | Belongs to the 62 kDa family role | secreted | 0.012 | 1.9 |
| gi|157115994  AAEL007394 | hypothetical protein [Aedes aegypti] | 18953.8 | 22 | 6 | 102 | | MLCMAYR | | --- | | MLCMAYR | | LGPMSYYR | | LGPMSYYR | | LGPMSYYR | | VGWMDKGTR | | NREVEFFSK | | SNSMYAFMDCTFIR | | SNSMYAFMDCTFIR | | SNSMYAFMDCTFIR | | SNSMYAFMDCTFIR | | TAELTEIEAMCNMEFR | | TAELTEIEAMCNMEFR | | Insect pheromone/odorant-binding proteins domain | secreted | 0.001 | +2.5 |
| gi|108883988  AAEL000732 | conserved hypothetical protein [Aedes aegypti] | 65165.2 | 25 | 29 | 1500 | | YGFMGLK | | --- | | VEEYQR | | YGFMGLK | | YGFMGLK | | VQIYNKK | | TQIEGLLK | | FLEEQKR | | GIKQDFEK | | DKLGDFYR | | DTIEWNLK | | RTQIEGLLK | | TEVDHLLVR | | TEVDHLLVR | | IESQFSDYK | | IESQFSDYK | | STIVFSTWTK | | STIVFSTWTK | | KTEVDHLLVR | | KTEVDHLLVR | | GYVKVEEYQR | | LTVVCNNYYK | | LTVVCNNYYK | | FDPHVPDEIPK | | FDPHVPDEIPK | | VVVADYGCTSFR | | VVVADYGCTSFR | | DINHLPHVLVAR | | DINHLPHVLVAR | | KLDEEVGTLHEK | | NALDHMFVSQMK | | NALDHMFVSQMK | | NALDHMFVSQMK | | NALDHMFVSQMK | | NALDHMFVSQMK | | CVVATGNDFDVHK | | NSDAKDTIEWNLK | | NSDAKDTIEWNLK | | VFGALVGDTSDFGSIR | | VFGALVGDTSDFGSIR | | DLCVTYLNSNQMSK | | DLCVTYLNSNQMSK | | DLCVTYLNSNQMSK | | CVVATGNDFDVHKDK | | CVVATGNDFDVHKDK | | RLEGEDSVLLEATVLK | | RLEGEDSVLLEATVLK | | TLNDELVELQQHTSEK | | TLNDELVELQQHTSEK | | LFEATLSSLPNSLTELR | | IVTPSGNPMSNVNLHLEG | | NSLHNYVATPK | |  | intracellular | 0.03 | 2 |
| gi|108878609  AAEL005672-PA | adenosine deaminase [Aedes aegypti] | 59947.8 | 26 | 28 | 1270 | | ENLWQK | | --- | | FIYAPGR | | FIYAPGR | | IGHGFAVLK | | IGHGFAVLK | | SLDDVWSK | | LKLEEMVK | | HIFEVLDR | | HIFEVLDR | | QYYHDSLK | | QYYHDSLK | | FATDDEFLK | | FATDDEFLK | | RIGHGFAVLK | | SNHPEFIGAK | | SNHPEFIGAK | | EWSLVSEIR | | EWSLVSEIR | | SLLEFAPALLK | | SLLEFAPALLK | | GDFGVSHGPQFK | | GDFGVSHGPQFK | | GVLPDVYDLDGK | | GVLPDVYDLDGK | | IGHGFAVLKHPK | | QWMTDKVYDAK | | QWMTDKVYDAK | | TDQNLIDAVLLGSK | | HIFEVLDRFGQSK | | TDQNLIDAVLLGSKR | | TDQNLIDAVLLGSKR | | QFYDDHVQYLEFR | | QFYDDHVQYLEFR | | QICIEINPISNQVLK | | QFYDDHVQYLEFR | | QFYDDHVQYLEFR | | QICIEINPISNQVLK | | RQICIEINPISNQVLK | | RQICIEINPISNQVLK | | QLALNSIEYSAMNSEEK | | VSEVFSLYNSDPLNAYK | | VSEVFSLYNSDPLNAYK | | FPTFLAGFDLVGQEDPG | | R | | KFPTFLAGFDLVGQEDP | | Adenosine deaminase-related growth factors (ADGF), a novel family of secreted growth-factors with sequence similarity to adenosine deaminase | intracellular | 0.028 | 2.1 |
| gi|108883990 | fibrinogen and fibronectin [Aedes aegypti] | 33896,5 | 27 | 8 | 402 | | MMMRLK | | --- | | MMMRLK | | MMMRLK | | MMMRLK | | GGWWYYR | | GGWWYYR | | IKHLLQEK | | FGGGWIVLMQR | | FGGGWIVLMQR | | FGGGWIVLMQR | | SISWVWFSTEKK | | SISWVWFSTEKK | | SCFGASLTGIWQNK | | YAGYDAFAVGPEEER | | YAGYDAFAVGPEEER | | GYGFSTYDNDDNGCSNQ | | YGR | | GYGFSTYDNDDNGCSNQ | | YGR | | Intracellular cell signalling processes  Fibrinogen is involved in blood clotting | secreted | 0.04 | 2.3 |
| gi|157103422 | antifreeze protein, putative [Aedes aegypti] | 18055.7 | 28 | 10 | 547 | | RFVCEQA | | --- | | LAVLDSEQK | | LAVLDSEQK | | WIAGMPDNK | | QQQVEELAQR | | QQQVEELAQR | | QQQVEELAQR | | QQQVEELAQR | | VELWIGASDLAR | | VELWIGASDLAR | | TAKVELWIGASDLAR | | FMWHPTGLDVSYSK | | FMWHPTGLDVSYSK | | FMWHPTGLDVSYSK | | LINWHWNDVVCASMR | | LINWHWNDVVCASMR | | DGYEHCVHLWYEPSR | | DGYEHCVHLWYEPSR | | EGKFMWHPTGLDVSYSK | | bind to ice and inhibit its growth in a noncolligative manner | secreted | 0.008 | 1.9 |
| gi|18568284 | venom allergen [Aedes aegypti] | 29619.2 | 31 | 7 | 154 | | HGFPQAAR | | --- | | HGFPQAAR | | CIFAHDK | | KCIFAHDK | | CIFAHDKCR | | CQTGQNPQYR | | CQTGQNPQYR | | HNELRAEIACGK | | HNELRAEIACGK | | YYLVCNYSMTNMIEEPIY | | salivary antigen-5 related protein | secreted | 0.009 | 4.1 |
